# EDTA and IAA Ameliorates Phytoextraction Potential and Growth of Sunflower by Mitigating Cu-Induced Morphological and Biochemical Injuries

**DOI:** 10.3390/life13030759

**Published:** 2023-03-10

**Authors:** Naila Shah, Muhammad Irshad, Anwar Hussain, Muhammad Qadir, Waheed Murad, Asif Khan, Muhammad Awais, Abdulwahed Fahad Alrefaei, Sajid Ali

**Affiliations:** 1 Department of Botany, Garden Campus, Abdul Wali Khan University Mardan, Mardan 23200, Pakistan; 2 Department of Botany, Government Girls Degree College, Lund Khwar, Mardan 23200, Pakistan; 3 Laboratory of Phytochemistry, Department of Botany, University of São Paulo, São Paulo l05508-090, Brazil; 4 Institute of Science and Technology, Department of Environmental Science, Kunming 650032, China; 5 Department of Zoology, College of Science, King Saud University, P.O. Box 2455, Riyadh 2455, Saudi Arabia; 6 Department of Horticulture and Life Science, Yeungnam University, Gyeongsan 38541, Republic of Korea

**Keywords:** Copper hyperaccumulation, stress mitigation, EDTA and IAA, sunflower

## Abstract

As an essential micronutrient, copper is vital for normal growth and development of plants, however, its accumulation in soil exerts a severe negative impact on the agronomic characteristics and yield of the crop plants. Phytoextraction is a low-cost method for restoring soil fertility and avoiding losses due to heavy metal contamination. We found that using EDTA and IAA together improved sunflower hyperaccumulation capacity. Sunflowers were cultivated under various levels of Cu (0 (control), 25, 50, and 75 mg/kg of soil) and treated with EDTA alone or combined with IAA. The results revealed that the amended treatment significantly enhanced the absorption and accumulation of Cu in the sunflowers. Furthermore, the various doses of Cu significantly reduced the root and shoot growth of sunflowers in a concentration-dependent manner by impairing the chlorophyll content, hormones (indole 3-acetic acid, salicylic acid, and gibberellic acid), flavonoids, phenolics, and antioxidant response. The injurious effect of Cu was reduced by the addition of EDTA alone, and the supplementation of IAA led to a significant restoration of shoot growth (~70%) and root growth (~13%) as compared to the plant treated with Cu alone. Moreover, significantly higher levels of chlorophyll content, GA_3_, endogenous IAA, and flavonoids were recorded, indicating the effectiveness of the treatment in ameliorating plant health. The results also showed considerable restoration of the catalase and ascorbate peroxidase activities in plants treated with EDTA and IAA. These results are suggestive that application of EDTA and IAA enhances the Cu absorption potential of sunflower and increases its tolerance to copper, which may not only serve as a better technique for phytoextraction of Cu, but also to bring Cu contaminated soil under cultivation.

## 1. Introduction

Heavy metals are part of the natural soil system; however, their high concentration accumulated by plants and animals due to agricultural malpractices and other anthropogenic activities poses a serious concern [1]. In plants and algae, copper (Cu) is a crucial micronutrient. Plants utilize copper, particularly in ATP synthesis, photosynthesis, CO_2_ assimilation, and as a vital component of several proteins. However, the overuse of Cu in industry and mining has increased its concentrations to toxic levels in ecosystems [2]. Cu above the threshold level can cause kidney and liver dysfunction, anemia, intestine and stomach irritation, hypertension, and nervous system problems. Cu-induced phytotoxicity leads to physiological stress and causes stunted growth and leaf chlorosis [3]. Due to the presence of malondialdehyde (MDA), which promotes bilayer lipid and protein peroxidation, Cu seriously harms plants through oxidation. Reactive oxygen species, such as hydroxyl radicals (OH), superoxide radicals (O_2_) and singlet oxygen (^1^O_2_), exhibit increases due to toxic Cu levels; however, OH, SOD, and POD are crucial antioxidants for the scavenging of ROS [4,5]. 

The copper content in the soil should be reduced in order to avoid Cu-induced phytotoxicity. In general, heavy metal removal from polluted soil involves physical, chemical, and biological methods [6]. Most chemical and physical methods, such as stabilization, solidification, vitrification, electrokinetics, soil washing, and vapour extraction, are, however, costly and ineffective [7,8,9,10]. Due to its extensive use and economic viability, phytoextraction, a green technique that allows the removal of soil contaminants, has emerged as a viable alternative. Fast-growing plants are used in an efficient and eco-friendly manner to remove heavy metals and other dangerous substances from contaminated soils and accumulate in harvestable portions. Exposure duration also impacts the reclamation of metal under certain conditions, i.e., metal accumulates actively as plants grow; however, after a certain growth period, the reclamation remains the same and the plant is unable to accumulate more metal [11]. More than 400 plant species from 45 distinct plant families, ranging from tropical to temperate regions, have been documented and claimed to be able to withstand and absorb heavy metals from soil. Heavy metals are absorbed from soil to shoots through roots, which depends on the species of plant, availability of heavy metals, growth stage and use of fertilizers [12].

Chelates, such as EDTA, lower the pH of soil solutions by forming complexes with heavy metals, thus increasing metal bioavailability and facilitating metal translocation from soil to root and then shoot. Limited amounts of chelators are required to boost metal absorption by plants [13].

Plant hormones have a crucial role in several important physiological processes [14]. Indole-3-acetic acid (IAA), a naturally occurring auxin, has the power to control a variety of aspects of plant growth, including vascular tissue differentiation, growth, and elongation, the production of lateral roots, apical dominance, and fruit formation and ripening. There have been numerous publications on the phytoextraction of various heavy metals from a variety of plant species using EDTA and IAA; however, there is limited literature on the synergistic effects of EDTA and IAA assisted phytoremediation [13,15].

As a novel study, this work was aimed at finding out the phytoextraction capacity of sunflowers in Cu-contaminated soils in the presence of EDTA and IAA. Moreover, the biochemical signatures of the sunflowers exposed to Cu and all other treatments were also investigated.

## 2. Materials and Methods

### 2.1. Preparation of Soil

The soil used throughout the experiment to grow the sunflowers was a sandy loam soil with composition of sand and clay was used with particle size of approximately 0.5 mm and 0.002 mm, respectively. The sand and clay were mixed with manure with the approximate ratio of 2:1:1 to make the plant growth medium, which was then used to prepare a sandy loam for improved sunflower development. Pots containing 5 kg of soil were maintained in the green house at the department of Botany, Abdul Wali Khan University in Mardan.

### 2.2. Experimental Design

Viable and healthy sunflower seeds were purchased and the surface of the seeds was disinfected by 70% ethanol, and the ethanol was then washed off with sterile distilled water. The experiment was in three factorial combinations, i.e., Cu concentrations (25, 50, and 75 mg/kg of soil in the form of CuCl_2_ as a bioavailable form of Cu), EDTA (5 mM (1.45 g/kg)), and foliar application of IAA (2.5 µM sprayed at intervals of 5 days until harvest), EDTA and IAA (in the same concentrations as used in the separate treatments) were used in combination with different levels of the selected metal. Each treatment consists of three replicates, and every replicate had three plants. The pots received a thorough watering of tap water every morning and evening. Several biochemical tests were run to determine the effect of copper concentration, IAA, and EDTA treatment on the agronomic and physiological characteristics of the host plant, as well as metal accumulation.

### 2.3. Morphological Parameters

The sunflowers roots and shoots were measured in centimeters (cm). The fresh and dry weights of the root and shoot were calculated and represented in grams using an analytical weight balance. To obtain their dry weight, the samples were oven dried at 80 °C.

### 2.4. Estimation of Chlorophyll Contents

A UV-visible spectrophotometer was used to quantify chlorophyll (Biochrom Libra S22) [16]. For the purpose of extracting chlorophyll, 0.5 g of fresh leaves were crushed with 80% acetone, and Whatman filter paper No. 42 was used for the filtration of mixture. Additional acetone was used to dilute the solution by about 1 mL (approximately 2 mL of acetone). Two wavelengths, 663 and 645 nm, were used to measure the optical density (OD) in comparison to a blank surface.

### 2.5. Estimation of Phytohormones

For estimation of IAA, the Salkowski reagent technique [17], and salicylic acid was measured using 1% iron chloride [18]. The gibberellic acid content was determined using a wheat endosperm assay [19].

### 2.6. Metabolite Determination

Total flavonoids were determined by the AlCl_3_ method [20]. Leaf samples of 0.5 g were homogenized using 80% ethanol (5mL) and kept for incubation for 24 h to achieve full flavonoid extraction in the shaker. After that, the mixtures were centrifuged for 15 min at 10,000 rpm at 25 °C. 

For the determination of total phenolics, 16 mL of ethanol was added to 1 g of crushed plant leaves. Centrifugation of the homogenates were performed at 10,000 rpm after being incubated at an increased temperature (between 20 °C and 80 °C) for 3 h. The supernatants were concentrated to 1 mL at 40 °C by using a rotary evaporator after being filtered through filter paper (Whatman No. 42). Resolving the concentrations in 10 mL distilled water allowed for the measurement of phenolics [20]. 

In order to extract proline content, the solutions were centrifuged at 10,000 rpm after being incubated for 24 h at 4 °C for 5 min following the protocol of Bates et al. [21].

### 2.7. Determination of Antioxidant Response

Catalase activity (CAT) and ascorbate peroxidase (APX) were used for the determination of antioxidant responses. The cleavage of H_2_O_2_ was determined for CAT activity using the procedure of Radhakrishnan and Lee [22]. Approximately 0.1 mL of supernatant, 0.4 mL of 3% H_2_O_2_, and 0.1 mM EDTA were added to 2.6 mL of 0.05 M phosphate buffer (pH 7). The drop in H_2_O_2_ was accompanied by a reduction in absorbance at 240 nm, which was quantified as M H_2_O_2_ min^−1^ cleavage.

To estimate APX in the leaves, the Asada [23] procedure was used. The reaction mixture consists of 0.1 mL ascorbic acid (0.5 mM), 0.6 mL PBS (50 mM, pH 7.0), 0.1 mL H_2_O_2_ (0.1 mM), and 0.2 mL leaf extract. A decrease in optical density was measured at 290 nm. Protein content was calculated for each extract using the technique of Bradford [24]. 

### 2.8. Estimation of the Copper in Plant Biomass

Oven dried 0.5 g samples were mixed with perchloric acid (HCLO_4_) and nitric acid (HNO_3_) in the ratio of 1:4 in order to prepare samples for metal analysis using an atomic absorption spectrophotometer. After cooling, the mixture was filtered, and the final volume of the mixture was raised to 25 mL by adding distilled water. Control plant samples were treated using the same method as the positive control solution. With the exception of the inclusion of sample, the blank solution was created in the same manner as the sample solution. For quantification of the copper contents in the biomass, Amin et al.’s [5] method was followed using atomic absorption spectrophotometer (Perkin Elmer 700) to determine selected heavy metals.

### 2.9. Data Analysis

The trials were carried out three times, with the treatment conditions for copper, copper/EDTA, copper/IAA, and copper/EDTA/IAA being separated from the data acquired from the factorial testing. The significance level of *p* ≤ 0.05 was determined using one way ANOVA followed by DMRT using SPSS Statistical Package version 21 (IBM, Armonk, NY, USA).

## 3. Results 

### 3.1. Effect of Copper on the Growth of Sunflower

**Figure 1:**
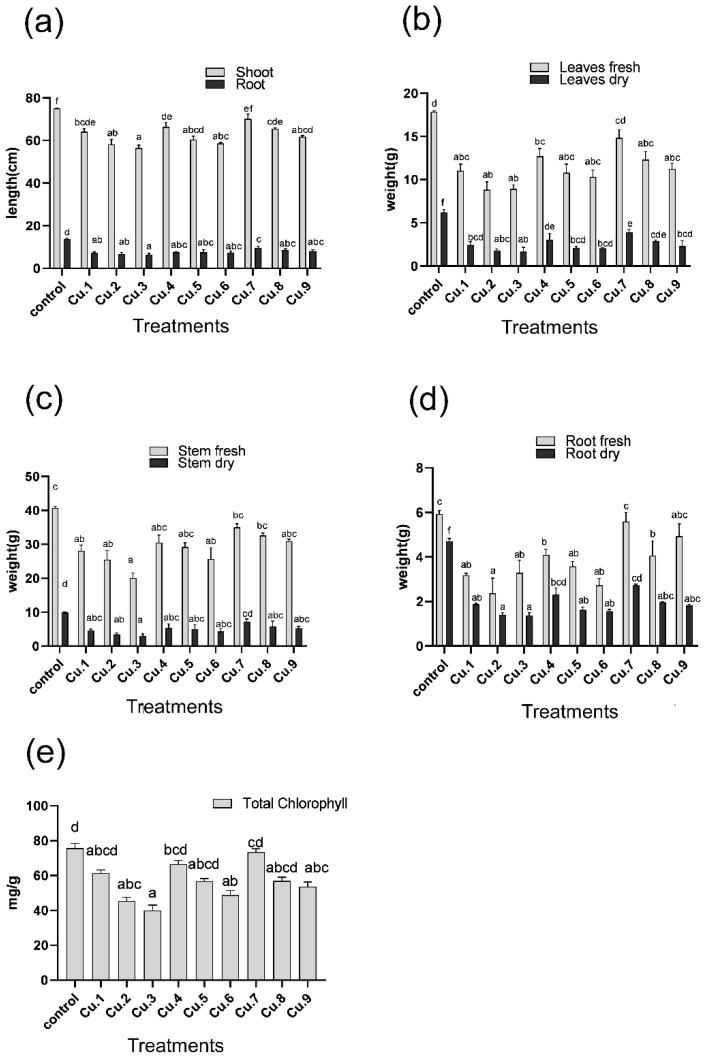
Effect of copper concentrations, EDTA and IAA application on (**a**) Root/shoot length, (**b**) leaves fresh/dry weight, (**c**) stem fresh/dry weight (**d**) root fresh/dry weight, and (**e**) total chlorophyll contents of the sunflower. Bars represent the means of the triplicates with standard error (±), and the letters indicate significant difference among treatments at the levels of *p* ≤ 0.05. Cu.1, Cu.2, and Cu.3: 25, 50 and 75 mg Cu/kg of soil, respectively; Cu.4, Cu.5 and Cu.6: 25, 50 and 75 mg Cu/kg of soil, respectively, with EDTA; Cu.7, Cu.8 and Cu.9: 25, 50 and 75 mg Cu/kg of soil, respectively, with EDTA and IAA.

Various copper concentrations led to a considerably decreased root and shoot length of the sunflowers as compared to the untreated control group (Figure 1a). At the highest copper concentration (75 mg Cu/kg soil), the greatest reduction in root and shoot length and of the fresh or dry weight of root, stem, and leaf was observed. A reduction of about 15%, 22%, and 25% in shoot length and 48%, 51%, and 53% in root length was recorded for 25, 50, and 75 mg Cu/kg of soil, respectively. In a concentration-based manner, fresh weight decreased by 57%, 68%, and 71%, and dry weight decreased by 34%, 43%, and 50%, respectively. Fresh and dry weight of different plant parts showed improvement with the application of EDTA along with different Cu concentrations (Figure 1b–d). 

A simultaneous addition of IAA and EDTA to different copper concentrations, exhibited an approximate increase of 8%, 10%, and 7% in shoot length and above 18%, 14%, and 13% in root length. Besides, an increase in root and shoot length was detected upon the foliar application of IAA. However, the positive results obtained after treating biomass with EDTA and IAA (23%, 19%, and 16%) show a declining pattern as the Cu level increases. 

### 3.2. Effect of Copper on Total Chlorophyll Contents

According to the results, the total chlorophyll contents in the leaves of sunflowers decreased by 19%, 41%, and 48% up to 75 mg Cu/kg soil (Figure 1e). However, improvement was noticed with the application of EDTA alone and in combination with IAA by 16%, 15%, and 18%, respectively.

### 3.3. Metal Accumulation and Translocation to Aerial Parts of the Host

**Figure 2:**
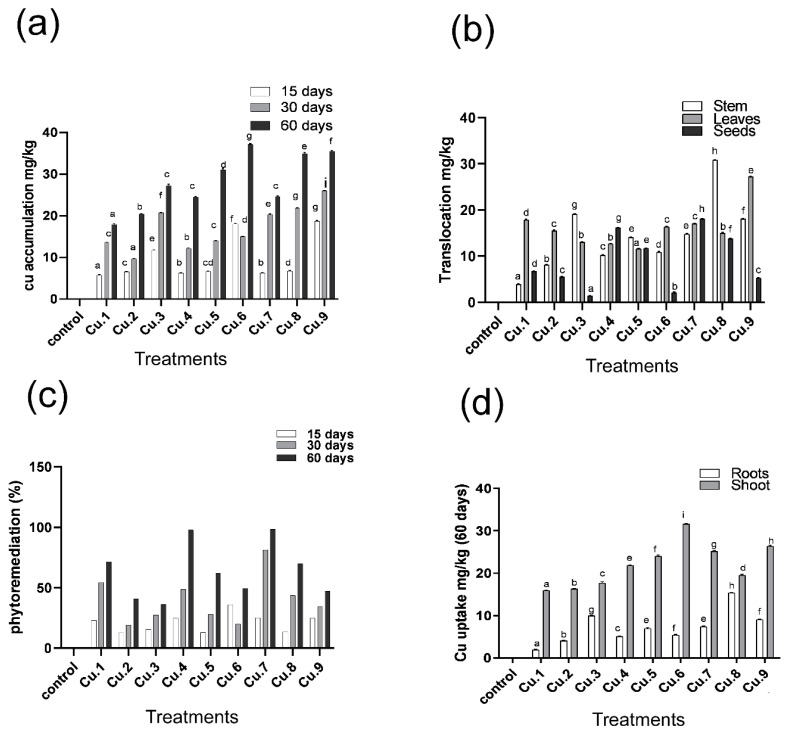
Effect of Cu as supplement, IAA and EDTA application on (**a**) Cu bioaccumulation, (**b**) Cu translocation, (**c**) phytoremediation %, and (**d**) root/shoot Cu absorption by sunflowers. Bars represent the means of the triplicates with standard error (±) and various letters indicates significant difference among treatments at the levels of *p* ≤ 0.05. Cu.1, Cu.2, and Cu.3: 25, 50 and 75 mg Cu/kg of soil, respectively; Cu.4, Cu.5 and Cu.6: 25, 50 and 75 mg Cu/kg of soil, respectively, with EDTA; Cu.7, Cu.8 and Cu.9: 25, 50 and 75 mg Cu/kg of soil, respectively, with EDTA and IAA.

The total copper content of various sunflower plant parts was determined using different concentrations of copper with EDTA alone and in combination with IAA. Copper accumulation in sunflowers increased as the levels of copper in the growth medium increased from 0 to 75 mg Cu/kg soil (Figure 2a–d). A similar pattern was observed when EDTA was applied to the soil, reflecting a rise with the elevation of Cu in the soil, which remained lower than that of plants untreated with EDTA. In the case of IAA application, high accumulation was noted with a rise in soil Cu. 

Similarly, the accumulation was directly proportional to exposure duration i.e., longer exposure times resulted in higher accumulation levels, and vice versa (Figure 2a). Cu accumulation was lower in the 15-day-old plant. An increase was recorded after 30 days of exposure. After 60 days of copper supplementation, significant accumulation was observed. Nonetheless, EDTA application in the soil improved copper accumulation, demonstrating a concentration-based increase with increased copper supplementation. 

The same patterns were observed after the transfer of metals to aerial parts of the plant (Figure 2b). The findings suggested that enhanced copper concentrations in the soil medium resulted in increases of copper hyperaccumulation in the aerial parts of the sunflowers. The distribution of copper in the aerial parts of the sunflower shows a pattern of stem > leaf > seed. EDTA application to the soil enhances the translocation capability of the stem, except for 50 mg Cu/kg soil, which recorded a higher accumulation than 75 mg Cu/kg of soil. Foliar IAA application also increases copper translocation; however, a declining trend was observed with a dose-dependent increase of the metal supplement in the soil. Contrarily, a concentration-based decline was noted in the translocation of the metal in the leaves (lowest in the case of 75 mg/kg). EDTA application in the soil and IAA foliar spray enhanced the translocation of copper to the leaves. In the case of translocation to the seeds, an increasing trend was recorded in copper accumulation, peaking at 75 mg Cu/kg soil supplementation. EDTA application resulted in a similar increasing pattern up to 50 mg Cu/kg soil; however, a dip was observed at 75 mg Cu/kg soil of metal spiked soil. With the foliar application of IAA, maximum accumulation was observed between 25 and 50 mg Cu/kg soil; however, a decline was recorded at 75 mg Cu/kg soil.

A decrease in the phytoremediation potential of copper was recorded with the concentration-based increase in metal supplementation (Figure 2c). The phytoremediation of the host increased and, in some cases, became twice as high after treating the soil with EDTA in the soil as compared to the control plants. A similar response was recorded with the foliar application of IAA, showing a similar increase with increasing metal concentration. A similar incline was recorded with the exposure duration. A higher percentage of phytoremediation of Cu was recorded in the plants exposed to 60 days of 25 mg Cu/kg soil of supplementation with EDTA amendment in the soil. A similar decline pattern was recorded with the inclination of soil metal supplement. EDTA application in the soil improved the metal uptake and its bioaccumulation, whereas the higher percentage of phytoremediation was noticed in most cases of Cu supplementation in comparison to the stressed treated control plants. Phytoremediation was improved, showing a similar declining trend, with the foliar spray of IAA.

Similarly, increasing the metal in a dose-dependent manner caused an increase in the root to shoot copper ratio (Figure 2d). The supplementation of IAA and EDTA resulted in an increase in metal accumulation in the roots as well as a similar increase in translocation. When compared to the roots of the sunflowers, maximum copper was accumulated in the shoots for all doses of copper with the application of EDTA alone and in combination with IAA.

### 3.4. Production of Phytohormones

**Figure 3:**
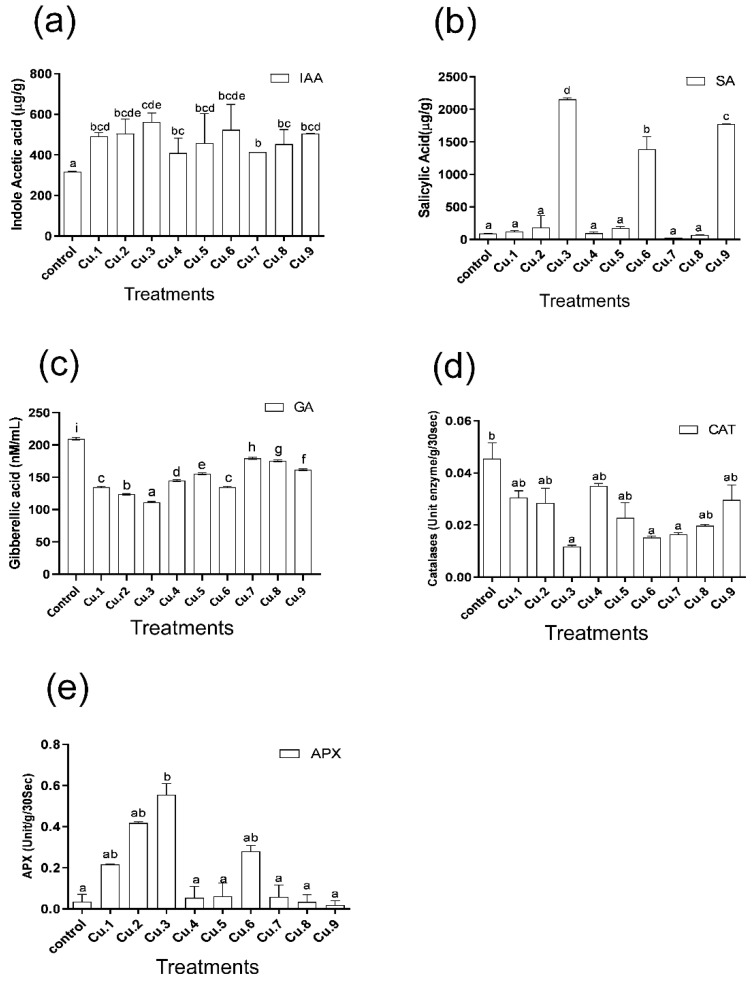
Effect of Cu supplementation, EDTA and IAA application on endogenous IAA (**a**), salicylic acid (**b**), GA_3_ (**c**), catalases (**d**), and ascorbate peroxidase (**e**) activity of sunflowers. Bars represent the means of the triplicates means with standard error (±), and the various letters indicates significant difference among treatments at the levels of *p* ≤ 0.05. Cu.1, Cu.2, and Cu.3: 25, 50 and 75 mg Cu/kg of soil, respectively; Cu.4, Cu.5 and Cu.6: 25, 50 and 75 mg Cu/kg of soil, respectively, with EDTA; Cu.7, Cu.8 and Cu.9: 25, 50 and 75 mg Cu/kg of soil, respectively, with EDTA and IAA.

Supplementing sunflowers with the aforementioned levels of copper resulted in a dose-dependent, significant production of endogenous IAA in the host plant, as documented after raising the Cu level in soil (Figure 3a). The use of EDTA and IAA also increased endogenous IAA production. The production of GA_3_ was severely reduced at all copper supplemented concentrations, indicating a concentration base decline (Figure 3b). GA_3_ production increased by applying EDTA and IAA as compared to untreated control plants. SA production displayed a contrary tendency to IAA production. No significant increase/decrease was reported at lower concentrations of the metal; however, an abrupt increase, an approximately 2200 µg/g of salicylic acid production, was recorded at 75 mg Cu/kg soil (Figure 3b). A similar effect was shown using either EDTA or IAA. Nonetheless, the improvement was much lower in comparison to stress control plants, i.e., only Cu supplemented plants. GA production was also reduced as the Cu levels increased (Figure 3c). The use of IAA and EDTA increased GA production, but the amount was lower when compared to untreated control plants with no Cu, EDTA, or IAA.

### 3.5. Response of Antioxidants

The plant antioxidant system increases in both primary and secondary stressful conditions. In the present study, plants treated with Cu supplements showed a decline in catalase production of 33%, 38%, and 74%, displaying the lowest value at 75 mg Cu/kg soil treatments (Figure 3c), whereas an improving rate of 10% was recorded at 25 mg Cu/kg soil with the supplementation of EDTA in the soil. However, the number of enzymatic units were less than that of the control plants. A different result was observed in the condition of ascorbic peroxidase, which increased multifold with the increase of metal in the soil, with higher enzyme units recorded at 75 mg Cu/kg soil (Figure 3d). Interestingly, an abrupt dip was recorded in the production of ascorbate peroxidase with EDTA application in soil, except for 75 mg/kg, which showed a significant increase. IAA foliar application with the previously mentioned Cu supplementation in the soil yielded the lowest value. 

### 3.6. Production of Metabolites

**Figure 4:**
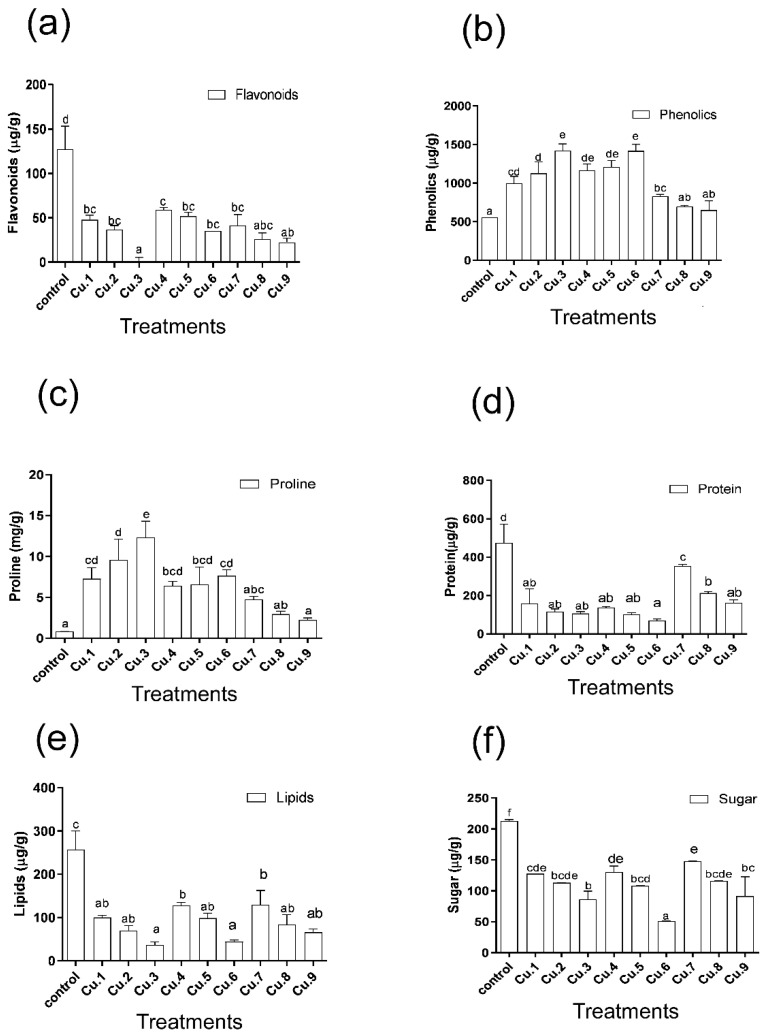
Effect of Cu supplementation, EDTA, and IAA application on flavonoids (**a**), phenolics (**b**), proline (**c**), protein (**d**), lipids (**e**) and sugar contents (**f**) of sunflowers. Bars represent the means of the triplicates with standard error (±) and various letters indicates significant difference among treatments at the levels of *p* ≤ 0.05. Cu.1, Cu.2, and Cu.3: 25, 50 and 75 mg Cu/kg of soil, respectively; Cu.4, Cu.5 and Cu.6: 25, 50 and 75 mg Cu/kg of soil, respectively, with EDTA; Cu.7, Cu.8 and Cu.9: 25, 50 and 75 mg Cu/kg of soil, respectively, with EDTA and IAA.

Cu treatment has a negative impact on endogenous flavonoid production in plants (Figure 4a). Decreases in the concentrations of the endogenous flavonoid contents were recorded (63%, 71%, and 76%) in a concentration-dependent manner, and the lowest was noted at 75 mg Cu/kg soil. EDTA applications in the soil boost flavonoid production by over 9%, 12%, and 4%, respectively, and foliar IAA applications boost flavonoid production in the host plant; however, the amount is lower when compared to untreated control plants. Furthermore, after supplementation with Cu, endogenous phenolics concentrations increased in the plants, indicating a positive relationship with Cu levels (Figure 4b). Similarly, EDTA application significantly increased phenolic production when compared to untreated control plants. For example, foliar application of IAA resulted in a decrease in total phenolics; however, endogenous phenolics were higher than in control plants. The higher the Cu levels, the greater the proline accumulation, and thus they show a direct relationship with Cu supplementation, similar to phenolic contents (Figure 4c). Supplementation of EDTA in the soil significantly decreased endogenous proline accumulation, but it was still greater than in the untreated plants. Foliar IAA resulted in a further dip, with the lowest level recorded at 75 mg Cu/kg soil. 

Total protein decreased significantly by 66%, 76%, and 77% and the lipids by 61%, 73%, and 86% in host plants under Cu stress (Figure 4d,e). The application of EDTA enhanced the protein content by 41%, 21%, and 11%, and the lipid content by 11%, 12%, and 12%; nevertheless, the quantity was much lower in relation to Cu treated plants, but an increase was noted with the IAA application. The same patterns were observed in the flowers endogenous total sugar contents, with decreases of 40%, 47%, and 60% with increasing Cu supplementation (Figure 4f). Increases of about 2%, 1%, and 2% were observed with EDTA in the soil and IAA foliar application, respectively; nevertheless, the quantities were lower when compared to the plants with no copper supplementation. 

## 4. Discussion

Pollution of the agroecosystem occurs because of anthropogenic activities, including the industrial use of certain chemicals and industrial effluents. The use of industrial water for irrigation of agricultural land leads to the buildup of certain hazardous chemicals, including cadmium (0.05 mg kg^−1^), chromium (98.94 mg kg^−1^), lead (1.35 mg kg^−1^), copper (8.44 mg kg^−1^), and others [25,26]. Among the metals, copper is an essential heavy metal that is extensively used in pesticides and insecticides, leading to the buildup of copper in agroecosystems. In the current study, different copper concentrations were used to assess the phytoextraction potential of sunflower in a controlled environment [27]. Excessive copper supplementation significantly reduced the sunflowers growth attributes in terms of root and shoot length, and fresh and dry biomass (*p* ≤ 0.05). Copper toxicity induces nutritional imbalances in plants and constrains their growth. This may have a paramount importance in the case of an essential plant macronutrient—phosphorus (P) [28]. Foliar application of IAA improved the agronomy of the plants in the presence of the mentioned copper concentrations. IAA has a 2-fold function: firstly, it acts as plant growth-promoting hormones and enhances host agronomic features and biomass production, while secondly, it was recently discovered that the IAA (and GA) have a role in stress mitigation. IAA and GA_3_ have long been known for their plant growth promotion and stress mitigation potential, and as a result of plant growth promotion, the size of the plant increases and the metal is distributed over a larger area, leading to stress mitigation [29]. Hence, in the current situation, IAA not only improves host growth and biomass production but also efficiently mitigate the copper stress in the host plant [30,31]. Similarly, results were also recorded in the case EDTA, showing improvements in the fresh and dry mass of the plants grown in the soil amended with copper supplementation. In a study with *Brassica napus*, the application of EDTA improved biomass of *B. napus* in a Cu-amended hydroponic system [32]. Our findings are in positive correlation with the previous studies, as the synergistic combination of IAA and EDTA significantly reduced Cu-induced toxicity in sunflower seedlings, possibly through reducing Cu-induced oxidative damage, and thus enhanced the morphological features of the tested plants [33,34,35,36]. In the case of chlorophyll contents, a decline was recorded up to 75 mg/kg of copper stress. The application of EDTA and IAA supplementation greatly increases the chlorophyll contents, which are, however, lower than those of untreated control plants [37,38]. 

By increasing the Cu concentration in the soil, the endogenous IAA content increased, which had a positive impact on plant growth promotion and stress mitigation. On the other hand, interesting results were recorded in the case of endogenous salicylic acid production. No significant increase or decrease was recorded at lower concentrations of the metal whereas a significant increase (approximately 2200 µg/g of salicylic acid production) was displayed at 75 mg Cu/kg soil. Similarly, either EDTA or IAA has no effect, except in the case of the 75 mg Cu/kg soil treatment, showing an abrupt increase in both cases. Plants release significant quantities of salicylic acid in order to mitigate the stress by activating several stress response genes, including heat shock protein, chaperon proteins, lower molecular weight osmolyte production, and several other mechanisms, in order to cope with the stressful environment [39]. Higher SA production helps the host grow normally in stressful conditions by maintaining its normal vigor. Because of the lower GA production, the host growth attribute was negatively regulated, resulting in lower biomass production and yield. The addition of EDTA and IAA regulated GA production positively, improving the host’s agronomic attributes [40,41]. Aside from growth and stress phytohormones, the release of flavonoids and phenolics, such as proline, lower the molecular weight, and sugar and protein maintain cellular viability and homeostasis [27,30,31,42]. With regards to metabolites, flavonoids are of prime importance, and in the presence of competitive inhibitors, all flavonoids are able to chelate copper; however, some compounds, particularly those containing the 3-hydroxyl group in association with the 4-keto group and the 2,3-double bond or possessing the 5,6,7-trihydroxyl substitution (baicalein), were very potent even in a highly competitive environment [41,43]. In this study, different concentrations of Cu negatively compressed the endogenous production of flavonoids in the host plant. Dose-dependent declines in the endogenous flavonoid contents were recorded, and the lowest was recorded at 75 mg Cu/kg soil. EDTA and IAA applications significantly enhanced flavonoid production.

Contrarily, the endogenous phenolics were increased in soil that was supplemented with Cu, showing a positive relationship to Cu levels. Previously, Cu stress stimulated the production of phenolics in *Colobanthus quitensis* [44], *Zea mays* [45] and *Phaeodactylum tricornutum* [46]. Similarly, *Dunaliella tertiolecta* was found to excrete almost double polyphenol concentration in the 790 nM L^−1^ copper enrichment experiment [47]. These studies show that in response to Cu stress, plants may stimulate the production of antioxidant secondary metabolites, mainly phenylpropanoids, which have a quenching effect against heavy metals. These phenolic compounds act as nonenzymatic antioxidants and directly quench the reactive oxygen species, thereby reducing their attack on biological membranes, and hence cells remain viable, ensuring their normal growth and development [48,49]. When compared to untreated control plants, EDTA application increases phenolic production even more. For example, foliar application of IAA resulted in a decrease in total phenolics; however, endogenous phenolics were higher when compared to control plants, i.e., plants without Cu treatments. Endogenous proline production was significantly reduced with EDTA application, but it remained higher than in control plants. In the case of IAA application, a further decrease was observed, with the level reaching its lowest point at 75 mg Cu/kg soil. The amounts of proline content at 75 mg Cu/kg soil were comparable to those of untreated control plants. Our results are in positive correlation with the findings of Saleem et al. [50] who reported improved *Corchorus capsularis* L. biomass and phenolics, and enhanced uptake of copper in response to EDTA treatments in Cu-augmented hydroponic solutions.

When the soil was enriched with Cu, the total lipids and proteins in the plants decreased significantly. The copper binds to certain enzymes and acts as a competitive inhibitor of the enzymes, inducing conformational changes that result in the decrease in protein content. The configurational changes make the enzyme either lower its optimal activity or, in some cases, cease its normal functions [51]. The abnormalities of these enzymes lead to severe physiological changes, including altered metabolite production, and abnormal glucose and lipid metabolism [52]. This altered glucose and lipid metabolism leads to lower glucose and lipid production [53]. Moreover, the plant absorbs these metals with an active expenditure of energy, and probably the majority of these energy compounds are wasted in the uptake of the metal, leading to their reduction and lower conversion to storage molecules, i.e., lipids [54,55]. EDTA application and foliar spray of IAA enhanced the protein and lipid contents; however, the amount was much lower in comparison to untreated control plants. The same patterns were observed in the host’s endogenous entire sugar content, which decreased as Cu supplementation increased. An increase in sugar was observed with EDTA and IAA foliar applications, nevertheless, the quantity was lower in comparison to plant with no supplementation of copper in the soil.

Plants produce a gamut of enzymatic antioxidants to cope with secondary oxidative stress due to environmental constraints [33,56]. Treating plants with Cu supplements reduces catalase production as metal supplementation increases in the soil; the lowest level was recorded at 75 mg Cu/kg soil treatments. This is probably due to the fact that catalases are more sensitive to copper stress, leading to conformational changes and degradation [57]. Enhancements were noted with EDTA and IAA-assisted applications; however, the number of enzyme units was lower as compared to control plants. An interesting contrast was observed with APX, showing a direct proportional and several-fold increase with copper supplementations in the growth medium, and higher enzyme units were observed at 75 mg Cu/kg soil, implying that Cu stimulates the oxidative capacity, which is responsible for the conversion of H_2_O_2_ to water and O_2_. APX is a component of the ascorbate-glutathione pathway, which plays a role in scavenging H_2_O_2_ [34,57]. Interestingly, an abrupt dip was recorded in the production of ascorbate peroxidase with the supplementation of EDTA in growth medium, except for 75 mg Cu/kg soil, which showed a significant increase. The lowest value was obtained with the foliar application of IAA in conjunction with the mentioned Cu supplementation in the soil [23].

As an essential micronutrient, plants tend to accumulate higher quantities of copper in their tissue [58]. Higher quantities of copper are taken up by plant roots with increases in metal levels and exposure times. The total copper content in plant biomass boosts with the inclination of metal concentration in the growth medium, showing a multifold accumulation from 0 to 75 mg Cu/kg soil. Similar results were previously recorded in the case of sunflower, brassica, and soybean treated with cadmium, chromium, and arsenic, showing a direct relation between metal levels and exposure duration [52,55,59,60]. A similar pattern was observed when EDTA was applied to soil. In the case of the IAA application, higher accumulation was also noted as soil metal supplementation increased. The plant spiked with 50 mg Cu/kg soil and treated with IAA foliarly had the highest accumulation. For instance, the copper accumulation was lower in the case of application of EDTA and IAA as compared to untreated plants with EDTA and IAA supplemented with the mentioned levels of copper [38]. 

Similarly, the accumulation increases with an increase in exposure time [61]. Plants exposed for 15 days to Cu stress showed lower accumulation, with the exception of 75 mg/kg of copper, which showed a decline with the passage of time. Except for the plants exposed to 50 mg Cu/kg soil with foliar application of IAA [59], for which an increase was observed after 30 days of copper exposure. A comparable rise was also documented in the case of 60 days of exposure to copper supplementation, showing an increase up to 50 mg Cu/kg soil; however, a decline was noted in the case of 75mg Cu/kg soil [62]. Nonetheless, EDTA application in the soil further improved the copper accumulation, showing a concentration-based increase with the increase of copper supplementing [63]. Foliar application of IAA, for example, increases copper accumulation, although a declining pattern was observed with the incline of metal in the soil [64]. The decline recorded with the duration was due to more copper accumulation and the subsequent harm due to metal stress and successive oxidative stress; it negatively affects the physiological attributes of the host by altering the physiology of the host, lowering the biomass, and, therefore, lowering the phytoremediation potential of the host plant [35,36,59,60]. Similar arrays were observed in the transfer of metals to the aerial parts of plants and were also documented in the case of the translocation of metals to the aboveground parts of the plants. Higher copper translocation was noted to the shoots of the plants, showing an increase up to 50 mg Cu/kg soil of the metal; however, a decline was recorded at 75 mg Cu/kg soil. EDTA and IAA applications promote the translocation capability of the host, resulting in higher accumulation with increasing copper in the soil, with the maximum accumulation recorded at 75 mg Cu/kg soil. The higher translocation to leaves and subsequent accumulation were probably due to the fact that plants shed their leaves early to get rid of the metal and its phytotoxicity. This is a strategy that plants mostly use to avoid the toxicity of the metal by translocating it to the leaves [6,65,66].

## 5. Conclusions

From the current study, it was evident that the *Helianthus annuus* L. is a hyperaccumulator that accumulates higher quantities of copper from polluted soil and effectively translocates it to the above ground plant parts, particularly leaves. Early shedding of the leaves was a strategy to get rid of the metal and subsequent toxicity. Moreover, the amount of copper supplementation and duration of exposure also increased the accumulation of metal which was positively improved with the application of EDTA and IAA.

## Data Availability

The datasets generated and/or analyzed during the current study are available from the corresponding authors upon reasonable request.

## References

[1] Amouei A., Cherati A., Naghipour D. (2018). Heavy metals contamination and risk assessment of surface soils of Babol in northern Iran. Health Scope.

[2] Roy R.N., Finck A., Blair G., Tandon H.L.S., AGL, FAO, Land and Water Development Division (2006). Plant nutrition for food security. FAO Fertil. Plant Nutr. Bull..

[3] Khator K., Shekhawat G.J.A.P.P. (2020). Cd-and Cu-induced phytotoxicity on 2–3 leaf stage of Cyamopsis tetragonoloba and its regulation by nitrate reductase and ROS quenching enzyme. Acta Physiol. Plant..

[4] Adrees M., Ali S., Rizwan M., Ibrahim M., Abbas F., Farid M., Zia-ur-Rehman M., Irshad M.K., Bharwana S.A.J.E.S., Research P. (2015). The effect of excess copper on growth and physiology of important food crops: A review. Environ. Sci. Pollut. Res..

[5] Zahoor M., Irshad M., Rahman H., Qasim M., Afridi S.G., Qadir M., Hussain A. (2017). Alleviation of heavy metal toxicity and phytostimulation of Brassica campestris L. by endophytic Mucor sp. MHR-7. Ecotoxicol. Environ. Saf..

[6] Qadir M., Hussain A., Hamayun M., Shah M., Iqbal A., Husna, Murad W. (2020). Phytohormones producing rhizobacterium alleviates chromium toxicity in Helianthus annuus L. by reducing chromate uptake and strengthening antioxidant system. Chemosphere.

[7] Liu L., Li W., Song W., Guo M. (2018). Remediation techniques for heavy metal-contaminated soils: Principles and applicability. Sci. Total Environ..

[8] Kogbara R.B. (2014). A review of the mechanical and leaching performance of stabilized/solidified contaminated soils. Environ. Rev..

[9] Fabbricino M., Ferraro A., Luongo V., Pontoni L., Race M. (2018). Soil washing optimization, recycling of the solution, and ecotoxicity assessment for the remediation of Pb-contaminated sites using EDDS. Sustainability.

[10] Wang Y., Li A., Cui C. (2021). Remediation of heavy metal-contaminated soils by electrokinetic technology: Mechanisms and applicability. Chemosphere.

[11] Raj D., Kumar A., Maiti S.K. (2020). *Brassica juncea* (L.) Czern. (Indian mustard): A putative plant species to facilitate the phytoremediation of mercury contaminated soils. Int. J. Phytoremediation.

[12] Saleem M.H., Ali S., Rehman M., Hasanuzzaman M., Rizwan M., Irshad S., Shafiq F., Iqbal M., Alharbi B.M., Alnusaire T.S.J.P. (2020). Jute: A potential candidate for phytoremediation of metals—A review. Plants.

[13] Shahid M., Austruy A., Echevarria G., Arshad M., Sanaullah M., Aslam M., Nadeem M., Nasim W., Dumat C.J.S., Journal S.C.A.I. (2014). EDTA-enhanced phytoremediation of heavy metals: A review. Soil Sediment Contam. Int. J..

[14] De Oliveira L.S., Brondani G.E., Molinari L.V., Dias R.Z., Teixeira G.L., Gonçalves A.N., de Almeida M. (2022). Optimal cytokinin/auxin balance for indirect shoot organogenesis of Eucalyptus cloeziana and production of ex vitro rooted micro-cuttings. J. For. Res..

[15] Ben Massoud M., Karmous I., El Ferjani E., Chaoui A.J.J.o.P.I. (2018). Alleviation of copper toxicity in germinating pea seeds by IAA, GA_3_, Ca and citric acid. J. Plant Interactions.

[16] Arnon D. (1949). Copper enzymes in isolated chloroplasts. Polyphenoloxidase in Beta vulgaris. Plant Physiol..

[17] Hussain A., Hasnain S.J. (2011). Interactions of bacterial cytokinins and IAA in the rhizosphere may alter phytostimulatory efficiency of rhizobacteria. World J. Microbiol. Biotechnol..

[18] Warrier R., Paul M., Vineetha M.J.G. (2013). Estimation of salicylic acid in Eucalyptus leaves using spectrophotometric methods. Environ. Sci. Biol..

[19] Ismail I., Hamayun M., Sayyed A., Din I., Gul H., Hussain A. (2016). Gibberellin and indole acetic acid production capacity of endophytic fungi isolated from *Zea mays* L.. Int. J. Biosci..

[20] El Far M.M., Taie H. (2009). Antioxidant activities, total anthocyanins, phenolics and flavonoids contents of some sweetpotato genotypes under stress of different concentrations of sucrose and sorbitol. Aust. J. Basic Appl. Sci..

[21] Bates C.J. (1977). Proline and hydroxyproline excretion and vitamin C status in elderly human subjects. J. Clin. Sci. Mol. Med..

[22] Radhakrishnan R., Lee I.-J. (2013). Spermine promotes acclimation to osmotic stress by modifying antioxidant, abscisic acid, and jasmonic acid signals in soybean. J. Plant Growth Regul..

[23] Asada K. (1992). Ascorbate peroxidase–a hydrogen peroxide-scavenging enzyme in plants. Physiol. Plant..

[24] Bradford M.M. (1976). A rapid and sensitive method for the quantitation of microgram quantities of protein utilizing the principle of protein-dye binding. Anal. Biochem..

[25] Pandey B., Suthar S., Singh V. (2016). Accumulation and health risk of heavy metals in sugarcane irrigated with industrial effluent in some rural areas of Uttarakhand, India. Process. Saf. Environ. Prot..

[26] Jamal A., Sarim M. (2018). Heavy metals distribution in different soil series of district Swabi, Khyber Pakhunkhawa, Pakistan. World Sci. News.

[27] Alengebawy A., Abdelkhalek S.T., Qureshi S.R., Wang M.-Q. (2021). Heavy metals and pesticides toxicity in agricultural soil and plants: Ecological risks and human health implications. Toxics.

[28] Feil S.B., Pii Y., Valentinuzzi F., Tiziani R., Mimmo T., Cesco S. (2020). Copper toxicity affects phosphorus uptake mechanisms at molecular and physiological levels in Cucumis sativus plants. Plant Physiol. Biochem..

[29] Mohamed H., Gomaa E. (2012). Effect of plant growth promoting Bacillus subtilis and Pseudomonas fluorescens on growth and pigment composition of radish plants (Raphanus sativus) under NaCl stress. Photosynthetica.

[30] Ismail A.H., Mehmood A., Qadir M., Husna A.I., Hamayun M., Khan N. (2020). Thermal stress alleviating potential of endophytic fungus rhizopus oryzae inoculated to sunflower (*Helianthus annuus* L.) and soybean (*Glycine max* L.). Pak. J. Bot..

[31] Hamayun M., Khan N., Khan M.N., Qadir M., Hussain A., Iqbal A., Khan S.A., Rehman K.U., Lee I.-J. (2021). Antimicrobial and plant growth-promoting activities of bacterial endophytes isolated from *Calotropis procera* (Ait.) WT Aiton. Biocell.

[32] Borker A.R., David K., Singhal N. (2020). Analysis of time varying response on uptake patterns of Cu and Zn ions under application of ethylene diamine disuccinic acid and gibberellic acid in Lolium perenne. Chemosphere.

[33] Qadir M., Hussain A., Hamayun M., Shah M., Iqbal A., Irshad M., Ahmad A., Lodhi M.A., Lee I.-J. (2021). Phytohormones Producing Acinetobacter bouvetii P1 Mitigates Chromate Stress in Sunflower by Provoking Host Antioxidant Response. Antioxidants.

[34] Husna, Hussain A., Shah M., Hamayun M., Iqbal A., Murad W., Irshad M., Qadir M., Kim H.-Y. (2021). Pseudocitrobacter anthropi reduces heavy metal uptake and improves phytohormones and antioxidant system in *Glycine max* L.. World J. Microbiol. Biotechnol..

[35] Husna, Hussain A., Shah M., Hamayun M., Iqbal A., Qadir M., Alataway A., Dewidar A.Z., Elansary H.O., Lee I.-J. (2023). Phytohormones producing rhizobacteria alleviate heavy metals stress in soybean through multilayered response. Microbiol. Res..

[36] Husna H., Hussain A., Shah M., Hamayun M., Iqbal A., Qadir M., Asim S., Lee I.-J. (2022). Stemphylium lycopersici and Stemphylium solani improved antioxidant system of soybean under chromate stress. Front. Microbiol..

[37] Posmyk M.M., Bałabusta M., Wieczorek M., Sliwinska E., Janas K. (2009). Melatonin applied to cucumber (*Cucumis sativus* L.) seeds improves germination during chilling stress. J. Pineal Res..

[38] Hayat K., Menhas S., Bundschuh J., Zhou P., Niazi N.K., Amna, Hussain A., Hayat S., Ali H., Wang J. (2020). Plant growth promotion and enhanced uptake of Cd by combinatorial application of *Bacillus pumilus* and EDTA on *Zea mays* L.. Int. J. Phytoremediation.

[39] Jumali S.S., Said I.M., Ismail I., Zainal Z. (2011). Genes induced by high concentration of salicylic acid in ‘Mitragyna speciosa’. Aust. J. Crop Sci..

[40] Raigond P., Buckseth T., Singh B., Kaundal B., Singh R.K., Singh B.P. (2019). Influence of Photoperiod and EDTA Salts on Endogenous Gibberellic Acid Concentration of Tissue Culture Grown Potato Microplants. Agric. Res..

[41] Ismaila A.H., Qadira M., Husnaa M.I., Ahmadb A., Hamayuna M. (2018). Endophytic fungi isolated from *Citrullus colocynthesl*. Leaves and Their potential for secretion of indole acetic acid and gibberellin. Appl. Environ. Biol. Sci..

[42] Rai K.K., Pandey N., Rai S.P. (2020). Salicylic acid and nitric oxide signaling in plant heat stress. Physiol. Plant..

[43] Říha M., Karlíčková J., Filipský T., Macáková K., Rocha L., Bovicelli P., Silvestri I.P., Saso L., Jahodář L., Hrdina R. (2014). In vitro evaluation of copper-chelating properties of flavonoids. RSC Adv..

[44] Contreras R.A., Pizarro M., Köhler H., Sáez C.A., Zúñiga G.E. (2018). Copper stress induces antioxidant responses and accumulation of sugars and phytochelatins in Antarctic *Colobanthus quitensis* (Kunth) Bartl. Biol. Res..

[45] Kısa D., Elmastaş M., Öztürk L., Kayır Ö. (2016). Responses of the phenolic compounds of *Zea mays* under heavy metal stress. Appl. Biol. Chem..

[46] Rico M., López A., Santana-Casiano J.M., Gonzàlez A.G., Gonzàlez-Dàvila M. (2013). Variability of the phenolic profile in the diatom Phaeodactylum tricornutum growing under copper and iron stress. Limnol. Oceanogr..

[47] Farid H.T. (2016). The effect of the marine cyanobacterium (*Trichodesmium erythraeum*) on iron speciation in seawater. Ph.D. Dissertation.

[48] Michalak A. (2006). Phenolic compounds and their antioxidant activity in plants growing under heavy metal stress. Pol. J. Environ. Stud..

[49] Surówka E., Hura T. (2020). Osmoprotectants and nonenzymatic antioxidants in halophytes. Handbook of Halophytes: From Molecules to Ecosystems towards Biosaline Agriculture.

[50] Saleem M.H., Ali S., Rehman M., Rana M.S., Rizwan M., Kamran M., Imran M., Riaz M., Soliman M.H., Elkelish A. (2020). Influence of phosphorus on copper phytoextraction via modulating cellular organelles in two jute (*Corchorus capsularis* L.) varieties grown in a copper mining soil of Hubei Province, China. Chemosphere.

[51] Secundo F. (2013). Conformational changes of enzymes upon immobilisation. Chem. Soc. Rev..

[52] Husna, Hussain A., Shah M., Hamayun M., Qadir M., Iqbal A. (2022). Heavy metal tolerant endophytic fungi *Aspergillus welwitschiaeimproves* growth, ceasing metal uptake and strengthening antioxidant system inGlycine maxL. Environ. Sci. Pollut. Res..

[53] Nonogaki K. (2000). New insights into sympathetic regulation of glucose and fat metabolism. Dibetologia.

[54] Juneja A., Ceballos R.M., Murthy G.S. (2013). Effects of environmental factors and nutrient availability on the biochemical composition of algae for biofuels production: A review. Energies.

[55] Qadir M., Hussain A., Shah M., Lee I.J., Iqbal A., Irshad M., Ismail, Sayyed A., Husna, Ahmad A. (2022). Comparative assessment of chromate bioremediation potential of *Pantoea conspicua* and *Aspergillus niger*. J. Hazard. Mater..

[56] Chandra J., Keshavkant S. (2021). Mechanisms underlying the phytotoxicity and genotoxicity of aluminum and their alleviation strategies: A review. Chemosphere.

[57] Wang S.-H., Yang Z.-M., Yang H., Lu B., Li S.-Q., Lu Y.-P. (2004). Copper-induced stress and antioxidative responses in roots of *Brassica juncea* L.. Bot. Bull. Acad. Sin..

[58] Ouzounidou G., Čiamporová M., Moustakas M., Karataglis S. (1995). Responses of maize (*Zea mays* L.) plants to copper stress—I. Growth, mineral content and ultrastructure of roots. Environ. Exp. Bot..

[59] Shah N., Qadir M., Irshad M., Hussain A., Hamayun M., Murad W., Khan A., Al-Harrasi A. (2022). Enhancement of Cadmium Phytoremediation Potential of *Helianthus annuus* L. with Application of EDTA and IAA. Metabolites.

[60] Qadir M., Hussain A., Shah M., Hamayun M., Iqbal A., Nadia (2022). Enhancement of chromate phytoremediation and soil reclamation potential of *Brassica campestris* L. by *Aspergillus niger*. Environ. Sci. Pollut. Res..

[61] Pratap H., Mamboya F., Mtolera M., Björk M. The effect of copper on the daily growth rate and photosynthetic efficiency of the brown macroalga *Padina boergesenii*. Proceedings of the Conference on Advances on Marine Sciences in Tanzania.

[62] Wu X., Yang Y., Liu H., Yue Z., Gao X., Yang F., Xing X. (2014). Effects of dietary copper supplementation on nutrient digestibility, serum biochemical indices, and growth rate of young female mink (Neovison vison). Czech J. Anim. Sci..

[63] Jiang B., Ma Y., Zhu G., Li J. (2020). Prediction of soil copper phytotoxicity to barley root elongation by an EDTA extraction method. J. Hazard. Mater..

[64] Mir A.R., Alam P., Hayat S. (2022). Auxin regulates growth, photosynthetic efficiency and mitigates copper induced toxicity via modulation of nutrient status, sugar metabolism and antioxidant potential in *Brassica juncea*. Plant Physiol. Biochem..

[65] Ng C.C., Rahman M.M., Boyce A.N., Abas M.R. (2016). Heavy metals phyto-assessment in commonly grown vegetables: Water spinach (*I. aquatica*) and okra (*A. esculentus*). Springerplus.

[66] Tuteja N., Gill S.S., Tuteja R. (2011). Plant responses to abiotic stresses: Shedding light on salt, drought, cold and heavy metal stress. Omics and Plant Abiotic Stress Tolrance.

